# Cardiac metabolism in the elderly: effects and consequences

**DOI:** 10.18632/aging.206071

**Published:** 2024-08-19

**Authors:** Mohammad Kasim Fatmi, Nadiyeh Rouhi, Lucian Lozonschi, Ji Li

**Affiliations:** 1 Nova Southeastern University Kiran C. Patel College of Osteopathic Medicine, Fort Lauderdale, FL 33328, USA; 2 Department of Surgery, University of South Florida, Tampa, FL 33612, USA; 3 Department of Physiology and Biophysics, University of Mississippi Medical Center, Jackson, MS 39110 USA; 4 Division of Cardiothoracic Surgery, Department of Surgery, Morsani College of Medicine, University of South Florida, Tampa, FL 33612, USA

**Keywords:** metabolism, aging, heart failure, Pdk4

Coronary artery disease (CAD) and heart failure (HF) are immensely prevalent in the aging population, contributing to their status as the world’s leading causes of death [1]. As the heart ages, its ability to recover from the stresses of myocardial infarction diminishes, leading to significant alteration in energy utilization and eventual heart failure [2]. Understanding the mechanisms behind these effects is key for developing new targeted therapies. We aim to explore the relationship between cardiac metabolism dysfunction and heart failure, emphasizing the need of innovative treatments to manage these interconnected cardiovascular conditions.

Following myocardial infarction (MI), the aged heart undergoes significant metabolic remodeling impacting its ability to contract and provide adequate cardiac output [3]. It’s understood that during the anaerobic conditions of MI, the heart shifts from fatty acid oxidation (FAO) to less efficient glucose oxidation [3]. Our murine study revealed that aged left ventricle tissue continues to rely on glucose oxidation post-MI, regulated by Pyruvate Dehydrogenase Kinase 4 (Pdk4) compared to their young counterparts [4]. This maladaptation in the left ventricle cardiomyocytes post-MI signifies a reduced capacity for the aged heart to recover cardiac output, progressing toward HF.

Targeting the macrophage migration inhibitory factor (MIF) signaling cascade, which modulates inflammation and metabolism, is a promising approach of responding to ischemic stress. Pdk4 regulates the pyruvate dehydrogenase complex (PDC), which is a critical enzyme complex in cellular metabolism. PDC controls the conversion of pyruvate to acetyl-CoA, thus linking glycolysis to the tricarboxylic acid (TCA) cycle and oxidative phosphorylation [5]. By phosphorylating and inhibiting PDC, Pdk4 decreases the conversion of pyruvate to acetyl-CoA, favoring glycolysis over oxidative phosphorylation. In the aged heart, decreased Pdk4 expression leads to a shift from FAO to chronic glucose oxidation, which is less efficient in ATP production. This metabolic shift is particularly detrimental post-MI, as the energy demand of the heart is not met, exacerbating cardiac dysfunction and contributing to HF.

Our research has found that MIF, like Pdk4, is diminished in the aged heart, reducing its adaptive response [6]. Upregulation of Pdk4 has been shown to enhance FAO and improve cardiac output in aged myocardium, suggesting a potential therapeutic target for post-MI cardiac dysfunction. Exploring treatment with agonistic MIF20 restores MIF signaling post-ischemia, minimizes infarction, and revitalizes cardiomyocyte metabolism by modulating Pdk4. MIF20 was found to modulate the maladaptation of Pdk4, allowing aged cardiomyocytes to recover to FAO and improve cardiac output [6]. The clinical relevance of protein therapies like MIF20 could be indispensable in post-MI treatment. This showcases a significant step in the right direction; however, further research is needed to develop effective pharmaceutical approaches to protein therapies that could reverse the impact of MI and prevent the progression of HF.

The interplay between Pdk4 and MIF in the aging heart provides a nuanced understanding of metabolic dysregulation post-MI. Targeting Pdk4 to restore efficient energy production through FAO and using MIF agonists to enhance adaptive responses represent promising therapeutic avenues. Ongoing research focuses on developing pharmacological agents that can precisely modulate these pathways. Such treatments have the potential to revolutionize post-MI care, particularly in elderly patients, by addressing the root causes of metabolic dysfunction and improving cardiac resilience to ischemic stress.

The consequences of MI are dire and addressing them requires a multifaceted approach. In addition to balancing cardiac metabolism following MI, another factor we must examine is the size of infarction. Previous studies found a strong correlation between deficient cardiomyocyte metabolism and infarction size. Investigating the administration of high dose metformin during reperfusion was found to lessen the infarct size, improve contractility, and even help modulate cardiomyocyte metabolism in humans and mice [7, 8]. Research into non-invasive treatments, such as cardiac stem cell (CSC) therapy, has demonstrated similarly promising results in reducing infarct size and enhancing cardiac output [9]. However, significant challenges remain in the clinical application of CSC therapy, necessitating further investigation into non-invasive clinical treatments. Reducing the size of the infarct is imperative for restoring cardiac homeostasis that subsequently improves cardiac metabolism.

Beyond the protein therapy and pharmacological interventions discussed, the field of post-MI cardiac repair is brimming with promise. Novel approaches like CRISPR gene editing for correcting genes linked to heart failure and exosome therapy for promoting tissue repair are actively being investigated. Clinical trials are also underway for stem cell therapy to regenerate damaged heart tissue. These advancements hold immense potential for improving cardiac function and patient outcomes, although further research is needed to determine their long-term safety and efficacy.

Mitigating the results of MI is essential to improving health outcomes for patients with CAD. Left ventricular systolic dysfunction (LVSD) and HF are considered the most common consequences for patients after an MI [10]. While invasive treatments exist to treat moderate to severe LVSD, they do not come without further complications. Coronary artery bypass grafting in patients with advanced CAD has been found to improve left ventricle dysfunction; however, right ventricular function often continues to decline, contributing to worsening HF [11]. Chronic heart failure, which often arises from a myocardial infarction, presents many complications, particularly for elderly patients as it advances to end-stage.

The incidence of CAD continues to rise worldwide, solidifying it as a significant public health concern. Preventing the progression to heart failure (HF) is critical for extending and enhancing the quality of life for patients with CAD. This goal can be achieved by addressing metabolic maladaptation and minimizing infarct size, both of which are vital in sustaining cardiac function as well as preventing the deterioration that leads to chronic HF. The importance of further research into potential treatments to correct cardiac dysfunction cannot be overstated. Innovations in areas such as protein therapy and pharmacological interventions hold immense promise for significantly improving energy production post-MI. As we advance our knowledge and evolve technology, these research efforts are essential for preserving the hearts we have and improving overall health outcomes worldwide.

In summary, detailed mechanistic insights into Pdk4 and MIF highlight their critical roles in cardiac metabolism and their potential as therapeutic targets for improving cardiac outcomes in the elderly post-MI (as shown in Figure 1). Future studies should aim to refine these therapeutic approaches and evaluate their efficacy in clinical settings.

**Figure 1:**
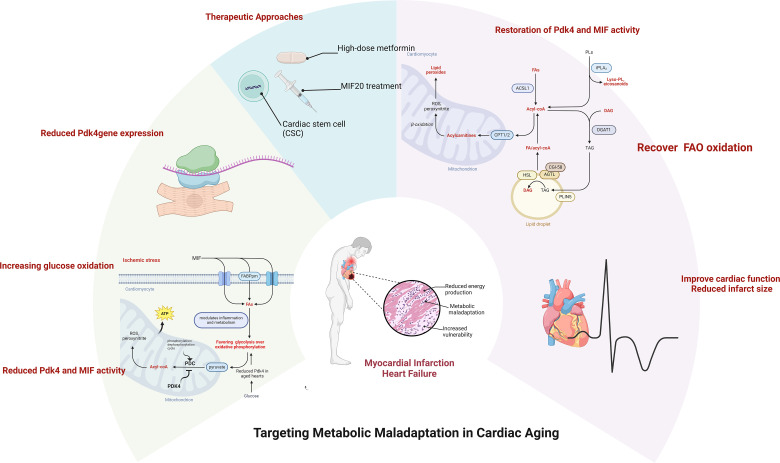
** Alterations in cardiac metabolic homeostasis occurred with aging.** The capability of metabolic adatpive response to pathological stress conditions declines in the aged heart. The pharmacologica and non-pharmacological approaches could rescue the aging-related vulnerability to the pathological challenge through an appropriate metabolic regulation. Created with Biorender.com.

## References

[1] Brown JC, et al. Treasure Island (FL): StatPearls Publishing. 2024.

[2] Jenča D, et al. ESC Heart Fail. 2021; 8:222–37. 10.1002/ehf2.13144 33319509 PMC7835562

[3] Jiang M, et al. Front Cardiovasc Med. 2021; 8:789267. 10.3389/fcvm.2021.789267 34957264 PMC8695728

[4] Fatmi MK, et al. Aging Cell. 2023; 22:e13800. 10.1111/acel.13800 36797808 PMC10086528

[5] Thorp EB. J Clin Invest. 2023; 133:e171953. 10.1172/JCI171953 37712418 PMC10503791

[6] Wang H, et al. Metabolism. 2024; 153:155792. 10.1016/j.metabol.2024.155792 38232801 PMC10932879

[7] Bates L, et al. Aging Dis. 2023; 14:1488–91. 10.14336/AD.2023.0205 37196121 PMC10529738

[8] Li Z, Wang H, et al. Biochem Biophys Res Commun. 2023; 659:46–53. 10.1016/j.bbrc.2023.04.004 37031594 PMC10190118

[9] Sugiura T, et al. Int J Mol Sci. 2024; 25:5772. 10.3390/ijms25115772 38891960 PMC11171475

[10] Cleland JG, et al. Heart. 2005 (Suppl 2); 91:ii7–13. 10.1136/hrt.2005.062026 15831613 PMC1876349

[11] Lozonschi L, et al. Asian Cardiovasc Thorac Ann. 2017; 25:586–93. 10.1177/0218492317744472 29153000

